# Supplementary material for: Extent of residual loss and damage from cyclones at household level: Evidence from the east coast of India
Source: Camb Prism Coast Futur. 2026 Apr 20;4:e9. doi: 10.1017/cft.2026.10028 (PMC13107360; doi:10.1017/cft.2026.10028)
Supplement: Das supplementary material [file S2754720526100286sup001.docx]

**APPENDIX**

**Appendix Table A1:**  List of possible L&Ds due to cyclones and the assertion from the local community on whether they suffer such damage during storms

| Long List and description of possible L&D due to cyclones | Does your village suffer such impacts due to cyclonic storms? |
| --- | --- |
| ***Direct Economic L&D*** | |
| Loss of houses | Y |
| Loss of crops, livestock, and fish stock | Y |
| Reduction in the quality of crops cultivated,  livestock, and fish stocks | Y |
| Loss of the quality of the land | Y |
| Loss of productivity and biodiversity ( soil-nutrient) of land from degradation and land use | Y |
| Reduction in the water table | - |
| Damage to community ponds | Y |
| Loss (purchase) of water for drinking and irrigation purposes | Y |
| Loss of private assets and amenities (boats, tractors, furniture, etc.) | Y |
| Loss of public infrastructure and assets | Y |
| ***Indirect Economic L&D*** | |
| Loss of household income generated from agriculture | Y |
| Reduction in day labor income from farming and other occupations | Y |
| Excess expenditure on consumption goods like electricity, ice, etc. | Y |
| Increased medical expenditure | Y |
| Reduction in food security (the ability to produce crops for Sale and for consumption) | Y |
| Change in the ability to access land (Shifting  from ownership to renting) | Y |
| The cost involved in spending extra time to collect water | Y |
| Increase in food prices | Y |
| Increased prices of other materials (timber) | Y |
| Negative Impact on the service sector, trade, and business | Y |
| Loss of income from non-farming activities | Y |
| Impact on knowledge | Y |
| Expenditure (Private) on Insurance buying | Y |
| Expenditure on other Risk reduction measures | Y |
| ***Direct Non-Economic L&D*** | |
| Loss of human life | Y |
| Physical injury or ailment | Y |
| Reduction in quality of life | Y |
| Impact on the reproductive health of women | Y |
| Increased fire hazard threat | - |
| Impact on ecosystems and biodiversity | Y |
| Loss of wetlands | Y |
| Increased evaporation rate | - |
| Increased frequency of soil erosion | Y |
| Damage to the habitat of fisheries, wildlife, and several  Flora and fauna (extinction) | Y |
| Deteriorated water quality | Y |
| Deteriorated air quality | Y |
| ***Indirect Non-Economic L&D*** | |
| Reduced access to medicine services | Y |
| Increases susceptibility to diseases | Y |
| Mental distress, Physiological – social stress | Y |
| Trust and cooperation among the households | Y |
| Increasing crime rate | Y |
| Inequality in accessing resources (grazing land) | Y |
| Forced to change occupational structure | Y |
| Damage to cultural heritage sites | Y |
| Loss of traditions (dress, festivals, etc.) | Y |
| Migration to another place | Y |
| School absenteeism of children | Y |

### Note: Y means yes, and ‘-‘means no, not applicable, or not aware.

### Source: List prepared by Sibylle Kabisch and colleagues, Adelphi Research, for their study on Climate-related Loss & Damage in India, and the responses are collected by the author from FGDs.

Appendix Table A2: Monetary value of Direct and Indirect L&Ds from the 1999 super cyclone and Phailin in 2013 for the study area as reported by the households

| **Type of L&D from cyclones** | Description of L&D indicators | Methods used to calculate the value of the L&D | Value of the average L&Ds suffered per household (in INR) during | |
| --- | --- | --- | --- | --- |
|  |  |  | Super Cyclone (1999) | Phailin (2013) |
| **DIRECT** economic and non-economic | Damage to house/dwelling structure | Money spent on repairing the damaged house | 48200 (49051) | 37700 (66037) |
|  | Loss of all crops | Approximate value of the crops lost (quantity of crops lost as reported by households multiplied by the then prevailing market price) | 24992 (43427) | 34558 (42496) |
|  | Loss of horticulture crops coconut, mango, cashew, keoda, etc.) | Same as above | 10317 (18746) | 2123 (4158) |
|  | Loss of timber trees (casuarina, etc.) | Lost timber income because of the falling of young trees | 3350 (9187) | 83 (645) |
|  | Loss of productive agricultural land | Money spent to bring back the saline-inundated land to a cultivable state | 13133 (25295) | 767 (2670) |
|  | Damage to nets and boats | Market price of lost items | 8390 (19281) | 4878 (16499) |
|  | Utensils lost | Market price of lost items | 778 (1424) | 55 (232) |
|  | Ornaments lost | Market price of lost items | 1017 (4652) | 0 |
|  | Currency notes lost | Value of currency notes | 33 (181) | 0 |
|  | Loss of Seeds stored at home | Market price of lost items | 559 (1073) | 292 (761) |
|  | Loss of Food items (in raw form) stored at home | Market price of lost items | 2566 (3783) | 669 (1300) |
|  | Loss of livestock | Market price of lost items | 6762 (17827) | 535 (2071) |
|  | Expenditure to treat the livestock disease due to the cyclone | Money spent at the veterinary clinic | 220 (401) | 102 (100) |
|  | Loss of poultry | Market price of lost items | 572 (993) | 3 (5) |
|  | Extra expenditure due to poultry disease | Money spent to treat the sick poultry | 52 (143) | 5 (12) |
|  | Expenditure to treat the health effects (disease, psychological disorder, etc.) of family members | Medical expenditure on family members to treat after the cyclone effects | 2732 (10572) | 535 (2751) |
|  | Expenditure made to improve drinking water quality | Money spent to improve the water quality of wells. The study villages did not have a piped water connection, and used to depend on tube wells and bore wells for water needs. | 1970 (4040) | 870 (2273) |
|  | Amount of compensation that can restore the quality of life to pre-disaster days^¥^ | A subjective estimate that people gave to compensate them for the non-economic L&D and the hardships and stress they have undergone. | 133750 (319502) | 56758 (109565) |
|  | Loss of family members (Y/N) |  | Y | N |
| **INDIRECT** economic and non-economic | Loss of labor income (man-days lost) | Man-days lost multiplied by wage rate | 10979 (10080) | 4699 (4793) |
|  | Effect on monthly expenditure on food due to an increase in food prices | Difference in their food bills between pre- and post-cyclone months | 3088 (4073) | 1168 (1560) |
|  | Improved knowledge of disaster (Y/N) | Non-monetisable  items | Y (95%) | Y (83%) |
|  | Trust and cooperation among households have gone down (Y/N) |  | Y (53%) | Y (45%) |
|  | Loss of Traditions(Y/N) |  | Y (43%) | Y (23%) |
|  | More inequality in accessing the natural resources of the area(Y/N) |  | Y (30%) | Y (18%) |
| Recovery period as per the households | Years needed to reach the pre-cyclone state |  | 4.3 years (0.5 to 15) | 1.25 years (0 to 3) |
| **Sum of economic loss and damage** | | Sum of the monetized items | 273460 | 145681 |

Source: Author (based on information from the FGD and household survey)

¥ Please see question 3.1.2 in household questionnaire for the question asked to elicit this response.

Appendix Table A3: Monetary value of Direct and Indirect L&Ds from the 1999 super cyclone for different occupational groups of the study area as reported by the households

| **Type of L&D from cyclones** | Description of L&D indicators | Value of the average L&Ds suffered per household (in INR) during Super cyclone (1999) | | |
| --- | --- | --- | --- | --- |
|  |  | Farmers (doing agriculture) | Fishermen | Services in the primary sector (like agricultural labor, small shop owners in villages, etc.) |
| **DIRECT** economic and non-economic | Damage to house/dwelling structure | 50227 (45315) | 37692 (31332) | 51880 (59578) |
|  | Loss of all crops | 28273 (21107) | 2692 (7256) | 33700 (62194) |
|  | Loss of horticulture crops coconut, mango, cashew, keoda, etc.) | 16705 (23930) | 3500 (6997) | 8240 (16472) |
|  | Loss of timber trees (casuarina, etc.) | 2341 (4878) | 5577 (12589) | 3080 (10185) |
|  | Loss of productive agricultural land | 15273 (16759) | 1692 (4441) | 17200 (35001) |
|  | Damage to nets and boats | 1486 (3805) | 29131 (26253) | 3680 (16027) |
|  | Utensils lost | 1086 (1927) | 192 (434) | 812 (1170) |
|  | Ornaments lost | 1091 (4275) | 2308 (8230) | 280 (1061) |
|  | Currency notes lost | 45 (213) | 0 | 40 (200) |
|  | Loss of Seeds stored at home | 1144 (1493) | 0 | 334 (586) |
|  | Loss of Food items (in raw form) stored at home | 4542 (5179) | 1127 (2151) | 1574 (1917) |
|  | Loss of livestock | 8682 (14353) | 500 (1414) | 8328 (23934) |
|  | Expenditure to treat the livestock disease due to the cyclone | 391 (527) | 38 (138) | 164 (309) |
|  | Loss of poultry | 514 (909) | 577 (1151) | 622 (1015) |
|  | Extra expenditure due to poultry disease | 56 (149) | 42 (138) | 54 (147) |
|  | Expenditure to treat the health effects (disease, psychological disorder, etc.) of family members | 4520 (16896) | 985 (1497) | 2066 (4379) |
|  | Expenditure made to improve drinking water quality | 3368 (5336) | 200 (285) | 1660 (3403) |
|  | Amount of compensation that can restore the quality of life to pre-disaster days | 156909 (300445) | 47692 (48269) | 160239 (414009) |
|  | Loss of family members (Y/N) | Y | Y | N |
| **INDIRECT** economic and non-economic | Loss of labor income (man-days lost) | 8666 (5426) | 8592 (3501) | 14256 (14096) |
|  | Effect on monthly expenditure on food due to an increase in food prices | 2273 (2160) | 4738 (7914) | 2946 (1813) |
|  | Improved knowledge of disaster (Y/N) | Y (100%) | Y (95%) | Y (83%) |
|  | Trust and cooperation among households have gone down (Y/N) | Y (45%) | Y (53%) | Y (45%) |
|  | Loss of Traditions(Y/N) | Y (33%) | Y (43%) | Y (23%) |
|  | More inequality in accessing the natural resources of the area(Y/N) | Y (40%) | Y (30%) | Y (18%) |
| Recovery period as per the households | Years needed to reach the pre-cyclone state | 5 years (1 to 10) | 2 years (1 to 10) | 5years (1 to 15) |
| **Sum of economic loss and damage** | | 307592 | 147275 | 311155 |

Source: Prepared by author based on FGD and Household survey

Appendix Table A4: Monetary value of Direct and Indirect L&Ds from the 2013 Cyclone Phailin for different occupational groups of the study area as reported by the households

| **Type of L&D from cyclones** | Description of L&D indicators | Value of the average L&Ds suffered per household (in INR) during Cyclone Phailin (2013) | | |
| --- | --- | --- | --- | --- |
|  |  | Farmers (doing agriculture) | Fishermen | Services provider in the primary sector (like agricultural labor, small shop owners in villages, etc.) |
| **DIRECT** economic and non-economic | Damage to house/dwelling structure | 14773 (29074) | 95769 (80670) | 27680 (65682) |
|  | Loss of all crops | 14636 (17044) | 154 (554) | 69980 (217602) |
|  | Loss of horticulture crops coconut, mango, cashew, keoda, etc.) | 3809 (5894) | 923 (2289) | 1264 (2312) |
|  | Loss of timber trees (casuarina, etc.) | 0 | 0 | 200 (1000) |
|  | Loss of productive agricultural land | 1409 (3698) | 0 | 600 (2198) |
|  | Damage to nets and boats | 32 (149) | 22462 (30181) | 0 |
|  | Utensils lost | 0 | 165 (439) | 46 (163) |
|  | Ornaments lost | 0 | 0 | 0 |
|  | Currency notes lost | 0 | 0 | 0 |
|  | Loss of Seeds stored at home | 614 (1081) | 0 | 160 (494) |
|  | Loss of Food items (in raw form) stored at home | 1027 (1597) | 504 (677) | 440 (1228) |
|  | Loss of livestock | 950 (1251) | 0 | 185 (1123) |
|  | Expenditure to treat the livestock disease due to the cyclone | 50 (173) | 25 (72) | 65 (102) |
|  | Loss of poultry | 0 | 0 | 8 (15) |
|  | Extra expenditure due to poultry disease | 0 | 0 | 12 |
|  | Expenditure to treat the health effects (disease, psychological disorder, etc.) of family members | 184 (272) | 338 (370) | 946 (3976) |
|  | Expenditure made to improve drinking water quality | 1841 (3523) | 423 (571) | 248 (555) |
|  | Amount of compensation that can restore the quality of life to pre-disaster days | 51818 (107052) | 103539 (132126) | 36780 (95558) |
|  | Loss of family members (Y/N) | N | Y | N |
| **INDIRECT** economic and non-economic | Loss of labor income (man-days lost) | 3555 (3472) | 8962 (5297) | 3490 (4397) |
|  | Effect on monthly expenditure on food due to an increase in food prices | 684 (1074) | 1831 (1943) | 1250 (1619) |
|  | Improved knowledge of disaster (Y/N) | Y (90%) | Y (75%) | Y (83%) |
|  | Trust and cooperation among households have gone down (Y/N) | Y (45%) | Y (40%) | Y (45%) |
|  | Loss of Traditions(Y/N) | Y (33%) | Y (43%) | Y (23%) |
|  | More inequality in accessing the natural resources of the area(Y/N) | Y (40%) | Y (30%) | Y (18%) |
| Recovery period as per the households | Years needed to reach the pre-cyclone state | 1 year (1 to 3) | 2 years (0.25 to 3) | 1years (0.3 to 3) |
| **Sum of economic loss and damage** | | 95382 | 235095 | 143354 |

Source: Prepared by the author based on FGD and the Household survey

Appendix Table A5: Monetary value of L&Ds from the 1999 super cyclone, compensations received, private adaptations, and residual L&Ds for different occupational groups

|  |  | | Super cyclone (1999) | | |
| --- | --- | --- | --- | --- | --- |
| Sl. No. | Type of loss, expenditure, and receipts | | Farmer | Fisherman | Service provider |
| 1 | Total monetized L&D (taken from the last row of Appendix table A2) | | 307592 (USD 5047) | 147275 (USD 2416) | 311155 (USD 5105) |
| 2 | Compensation received from the government and/or NGOs | | 15678 | 159482 | 40755 |
| 3 | Damage covered by insurance | | 7727 | 0 | 3090 |
| 4 | Total compensation received (2+3) | | 23405 | 159482 | 43845 |
| 5 | The amount of uncompensated L&Ds borne by the household (1 - 4) | | 284187 | -12207 | 267310 |
| 6 | Percentage of uncompensated L&Ds (6/1*100) | | 92% | - 8% | 86% |
| 7 | Private adaptation or private replacement expenditure (money and labor time spent privately to bring back normalcy) by the time of the survey | Agricultural output | 20455 | 1692 | 7720 |
|  |  | Assets and amenities | 15864 | 27423 | 13180 |
|  |  | Purchase of livestock and Fishery | 9877 | 2462 | 2840 |
| 8 | Total private adaptations made | | 46196 | 31577 | 23740 |
| 9 | Total amount covered by compensation, insurance, and private adaptations (4+8) | | 49601 | 191059 | 67585 |
| 10 | Residual L&Ds on the household (1-9) | | 257991 (USD 4232) | -43784 (USD 718) | 243570 (USD 3996) |
| 11 | Percentage of residual L&Ds on the household (10/1) | | 84% | -30% | 78% |
| 12 | Annualized L&Ds on the household in 2014 prices | | 17199 (USD 282) | -2920 (-USD 48) | 16238 (USD 266) |

Source: Author (based on information from Table A3 and household survey)

Appendix Table A6: Estimates of Household-level compensated and uncompensated L&D from cyclone Phaili (2013) for different occupational groups (in INR and in prices as prevailed in the year 2014)

|  |  | | Phailin (2013) | | |
| --- | --- | --- | --- | --- | --- |
| Sl. No. | Type of loss, expenditure, and receipts | | Farmer | Fisherman | Service provider |
| 1 | Total monetized L&D (taken from the last row of Appendix table A3) | | 95382 (USD 1565) | 235095 (USD 3857) | 143354 (USD 2351) |
| 2 | Compensation received from the government and/or NGOs | | 5226 | 53161 | 13585 |
| 3 | Damage covered by insurance | | 3909 | 0 | 1904 |
| 4 | Total compensation received (2+3) | | 9132 | 53161 | 15489 |
| 5 | The amount of uncompensated L&Ds borne by the household (1 - 4) | | 86,250 | 181,934 | 127,865 |
| 6 | Percentage of uncompensated L$Ds (5/1) | | 90% | 77% | 89% |
| 7 | Private adaptation or private replacement expenditure (money and labor time spent privately to bring back normalcy) by the time of the survey | Agricultural output | 6955 | 2308 | 1460 |
|  |  | Assets and amenities | 5857 | 30462 | 5556 |
|  |  | Purchase of livestock and Fishery | 1955 | 2308 | 2600 |
| 8 | Total private adaptations made | | 14766 | 35077 | 9616 |
| 9 | Total amount covered by compensation, insurance, and private adaptations (4+8) | | 23898 | 88238 | 25106 |
| 10 | Residual L&Ds on the household (1 - 9) | | 71484 | 146857 | 118249 |
| 11 | Percentage of residual L&Ds on the household | | 75% | 62% | 82% |
| 12 | Annualized L&Ds on the household in 2014 prices | | 16680 (USD 274) | 34267 (USD 562) | 27591 (USD 453) |

Source: Author (based on information from Table A4 and household survey)

**Appendix Table A7: Two sample t-test for comparison of average damage suffered during the Super cyclone and Phailin**

| Occupational groups | Average damage (super cyclone) | Difference with t-values | Average Damage (Phailin) | Difference with t-values |
| --- | --- | --- | --- | --- |
| Farmers (n=22) | 307592 | 61300  (t = 0.59) | 94382 | -80171  (t = 1.37) |
| Fishers+service providers (n=38) | 246291 |  | 174552 |  |
| Fishers (n=13) | 147276 | -157588  (t = 1.32) | 235069 | 114782  (t = 1.69) |
| Farmers + service providers (n=37) | 304865 |  | 120287 |  |
| Service providers (n=25) | 302256 | 54209  (t = 0.52) | 143084 | -3553  (t = 0.06) |
| Farmers + fishers (n = 35) | 248047 |  | 146637 |  |

**Appendix Table A8: Pairwise comparisons of damage suffered by marginal linear predictions with different adjustments for significance level**

| **Damage suffered** by occupational groups | Difference of means | Adjustment methods | | | | | |
| --- | --- | --- | --- | --- | --- | --- | --- |
|  |  | Turkey | | Sidak | | Bonferroni | |
| Phailin cyclone |  | t-value | P>t | t-value | P>t | t-value | P>t |
| fishers vs. farmers | 140687 | 1.85 | 0.16 | 1.85 | 0.19 | 1.85 | 0.2 |
| service providers vs. farmers | 48702 | 0.76 | 0.72 | 0.76 | 0.83 | 0.76 | 1 |
| service providers vs. fishers | -91985 | -1.23 | 0.43 | -1.23 | 0.52 | -1.23 | 0.66 |
| **Super cyclone** |  |  |  |  |  |  |  |
| fishers vs. farmers | -160315 | -1.2 | 0.46 | -1.2 | 0.56 | -1.2 | 0.71 |
| service providers vs. farmers | -5336 | -0.05 | 0.99 | -0.05 | 1 | -0.05 | 1 |
| service providers vs. fishers | 154980 | 1.16 | 0.47 | 1.16 | 0.57 | 1.16 | 0.74 |

**Appendix Table A9: Pairwise comparisons of compensation received by marginal linear predictions with different adjustments for significance level**

| **Compensation received** by occupational groups | Difference of means | Adjustment methods | | | | | |
| --- | --- | --- | --- | --- | --- | --- | --- |
|  |  | Turkey | | Sidak | | Bonferroni | |
| **Phailin cyclone** |  | t-value | P>t | t-value | P>t | t-value | P>t |
| fishers vs. farmers | 47934*** | 5.57 | 0 | 5.57 | 0 | 5.57 | 0.00 |
| service providers vs. farmers | 8359 | 1.16 | 0.48 | 1.16 | 0.57 | 1.16 | 0.75 |
| service providers vs. fishers | -39576*** | -4.71 | 0 | -4.71 | 0 | -4.71 | 0.00 |
| **Super cyclone** |  |  |  |  |  |  |  |
| fishers vs. farmers | 143803*** | 5.57 | 0.00 | 5.57 | 0.00 | 5.57 | 0.00 |
| service providers vs. farmers | 25077 | 1.16 | 0.48 | 1.16 | 0.57 | 1.16 | 0.75 |
| service providers vs. fishers | -118727*** | -4.71 | 0.00 | -4.71 | 0.00 | -4.71 | 0.00 |

**FOCUS GROUP DISCUSSION**

**WELCOME**

Thanks for agreeing to be part of the focus group. We appreciate your willingness to participate.

**INTRODUCTION**

Moderator and Participants Introduction

**Purpose of the FGD**

- To discuss the impacts of cyclone on your life, livelihood
- To discuss the issues affecting agriculture and related sectors in your village
- To discuss the drivers that could cause these issues (i.e. exposure and sensitivities)
- To discuss a climatic driver, the extreme weather event - cyclones and its effects on agriculture, livestock, housing and human health.
- To discuss the solutions that have been implemented at village level and the constraints the village is facing to implement these or some other solutions

Your input will be a key to past (and potential future) loss and damage estimate due to cyclones for Odisha.

**Part - I: Issues and causes**

1. What are the main five agricultural issues your village is facing? How do you think these issues are impacting you and your family?
2. What are the main causes behind these agricultural issues? Do you think these agricultural problems are driven by cyclonic storms? If yes, how?
3. Have you been hit by a cyclonic event in the recent past years?

- What is the biggest change that has happened to you and your community because of cyclones?
- What are the socio-economic consequences of these changes? ( Do you think you/your village suffered such damage from cyclones (read out different potential impacts from storms)?
- What important actions you as a community member have taken to overcome those challenges, to mitigate them and to avoid risk? (Adaptation strategies)
- According to you which institution can ease these types of constraints?
- What kind of assistance was provided and was that a useful one? Did that assistance helped you to recover? (sustainability of assistance)

**Part II: L & D estimates and adaptation**

- To assess and quantify the total past and potential future losses from impacts related to agriculture, livestock, fishery, housing and human health.
- To assess and quantify the related adaptation measures (and relief received)
- To then quantify the past and potential future residual L&D

1. Do cyclones affect your agro - biodiversity? (crops – species and varieties, seed sources, pest and diseases)

- What do you think are the main causes of decline in production due to a cyclonic event? What are its consequences on the crop yields?
- Which are the major hampered crops and how much is the value of loss (as a % of your household income/annual production) due to decrease in production of crops?
- To what extent your current and future food security (survival) is at risk because of such agricultural loss? (rise in food prices, loss of agricultural land etc.)
- What amount of damage and type of damage a cyclonic event causes to your livestock and fishery?
- What are the different types of loss in fishery sector?
- What strategies you adopt to overcome that situation?

1. Compared to the time before cyclone, how prepared do you feel now to minimize the risk of your family and household assets? (Less, same as before, more)

- If there is a cyclonic event, what do you have on your part to contribute to your resilience? What amount of cost is imposed on you in undertaking those coping measures? (adaptation)
- Who is the main assistance provider to you and your community? What more can be done by the government to overcome the residual loss and damage?
- How would you rate your ability to recover if any disaster with similar intensity comes in future? (low, moderate, very strong)

Let’s summarize some of the key points from our discussion. Is there anything else?

Thank you for taking out your time and providing your valuable input to this research project.

**Understanding and Quantifying Loss and Damages (L&Ds) from Cyclonic Storms in Odisha**

**Household Level Questionnaire**

Questionnaire Serial Number/Name of the Village:__________________________________

Name of the Panchayat/Taluka/Block/District:­­­­­­­­­­­­______________________________________

Cyclonic Storms: Yes/ No

**Section I: Details about Household**

- 1. **Details about Respondent and Household Head:**

| - - 1. Name of the Respondent: |  |
| --- | --- |
| - - 1. Mobile Number: |  |
| - - 1. Address: |  |
| 1.1.4 Age of the Respondent | _________Years |
| 1.1.5 Sex of the Respondent | Male/Female |
| 1.1.6 Are you head of the Household | Yes/ No |
| - - 1. Educational qualification of HH | Primary/ Higher Secondary/ College/ Post Graduation/ Technical Education/ Illiterate |
| - - 1. Occupational status of HH | Agriculture/ Fishing/ Others |
| - - 1. Years of experience in agriculture-related activities | ___________Years (approx.) |
| - - 1. Duration of stay at the present address | 0-10 Years/ 10-20 Years/ 20-50 Years/ > 50 Years |
| - - 1. Major source of income of the household | Agriculture/ Fishing/ Others |
| - - 1. Status of poverty of the HH | BPL/ APL/ AAY |

- 1. **Assets and Amenities:**

| - - 1. Type of dwelling unit | Pucca/ Semi-Pucca/ Kutchha |
| --- | --- |
| - - 1. Is this house owned by you? | Yes/ No |
| - - 1. What type of roofing material does your house have? | Concrete or cement/ Bricks/ Mud/ Thatch/ Iron Sheet/ Tile/ Asbestos Sheet/ Other |
| - - 1. Does your house have electricity? | Yes/ No |
| - - 1. Ownership status of agricultural land: | _________ha/ acre/…… |
| - - 1. Value of assets and amenities (approx.) | Agricultural :__________(in Rs.)  Non-agricultural :___________(in Rs.)  Total :_______________(in Rs.) |

- 1. **Income, Consumption Expenditure, Borrowing & Saving:**

| 1.3.1 Total income from different sources (Yearly) | Agriculture :____________ (in Rs.)  Fishing :________________ (in Rs.)  Livestock : _____________(in Rs.)  Others :________________(in Rs.) |
| --- | --- |
| 1.3.2 Consumption Expenditure (month-wise) | Food :______________(in Rs.)  Non-Food :____________(in Rs.) |
| - - 1. Have you purchased agricultural insurance in the last five years? | Yes/ No |
| - - 1. If yes, what type: |  |
| - - 1. Coverage of insurance: | Cyclonic Storms/ Heat Waves / Others |
| - - 1. If not purchased, please specify the reasons |  |
| - - 1. If Yes, do you purchase every year or occasionally | Every year/ Occasionally |
| - - 1. Do you have any outstanding borrowing? | Yes/ No |
| - - 1. If yes, please specify the reason for borrowing? |  |
| - - 1. If yes, how many loan you have taken? | _____(no.) |
| - - 1. Current amount of outstanding loan that was taken, by sources? | Total : ______(in Rs.)  Formal Institutions :_________(in Rs.)  Informal Institutions :_________(in Rs.) |
| - - 1. Are you regularly repaying the loan? | Yes/ No |

**Section II: Households’ Perception on Frequency and Intensity of Extreme Events (Cyclonic Storms) and Slow-onset Disasters (Heat Waves)**

| - - 1. Have you observed any change in the frequency and intensity of cyclonic storms over the years? | Yes/ No |
| --- | --- |
| If Yes, answer the following questions |  |
| - - 1. Frequency of cyclonic storms | Increase/ Decrease/ No Change/ Don’t know |
| - - 1. Intensity of cyclonic storms: | Increase/ Decrease/ No Change/ Don’t know |
| - - 1. How large do you feel is the effect of cyclonic storms on you and your family? | Very High/ High/ Medium/ Low/ No Impact |

| - - 1. Impacts of Cyclones | Super Cyclones  (1999) | Phailin  (2013) |
| --- | --- | --- |
| Affected (Yes/ No) |  |  |
| Level of Overall impact (Very High/ High/ Moderate/ Low/ No Impact) |  |  |
| Impact on livelihood (Very High/ High/ Moderate/ Low/ No Impact) |  |  |
| Major problems faced |  |  |

| - - 1. Have you observed any impact due to heat waves? | Yes/No |
| --- | --- |
| - - 1. Does it have impact on agriculture? | Yes/ No |
| - - 1. If yes, how will you describe the level of impact due to heat waves? | Very High Impact/ High/ Moderate/ Low/ No Impact |
| - - 1. What are other effects of heat waves? | Drinking water: Yes/ No  Health: Yes/ No  Livestock: Yes/ No  Fishing: Yes/ No |

**Section III: Loss and Damages (L&Ds) from Cyclonic Storms**

- 1. **Cyclonic Storms (3.1.1 are asked twice, once for super cyclone and then for Phailin)**

3.1.1 Loss and Damages from Cyclonic Storms (Super Cyclones/ Phailin) (per event)

Direct L&D Indicators - Economic / Non – Economic

| L&D Indicators – Direct | Intensity* | Units (describe) | Loss in monetary terms (income lost, value of assets lost, repair cost, expenditure made to recover, etc.) (approx) | Remarks |
| --- | --- | --- | --- | --- |
| Damage to house/ dwelling structure |  |  |  |  |
| Loss of agricultural crops |  |  |  |  |
| Loss of ag. land to saline inundation (permanent) |  |  |  |  |
| Loss of ag. land quality (can be recovered) |  |  |  |  |
| Damage to nets and boats |  |  |  |  |
| Damage to other properties/ assets |  |  |  |  |
| Loss of livestock |  |  |  |  |
| Impact on the health of livestock |  |  |  |  |
| Impact on the health of family members |  |  |  |  |
| Deteriorated water quality |  |  |  |  |

* Intensity - Very High Impact/ High/ Moderate/ Low/ No Impact

Indirect L&D Indicators - Economic / Non – Economic

| L&D Indicators – Indirect | Whether suffered (Y/N) | Intensity* | Monetary loss (In Rs.) (approx) | Remarks |
| --- | --- | --- | --- | --- |
| Loss of day labour income |  |  |  |  |
| Increase in food prices |  |  |  |  |
| Impact on Knowledge |  |  |  |  |
| Trust and cooperation among households |  |  |  |  |
| Loss of Traditions |  |  |  |  |
| Inequality in accessing resources |  |  |  |  |

* Intensity - Very High Impact/ High/ Moderate/ Low/ No Impact

3.1.2. How many years are required to revert back to normal activities after a cyclonic storm? __________ (may use agricultural activities or other activities as reference)

3.1.3. Along with economic losses, your family has suffered losses that cannot be compensated and also has undergone stress and hardships. If you are compensated, how much money will make you forget those sufferings or will leave you at a state similar to the pre-cyclone state. ----

**Section IV: Coping/ Adaptation to Cyclonic Storms**

- 1. Please mention the coping/ adaptation measures undertaken by you to reduce impacts on your livelihoods from cyclonic storms and mention the cost of each adaptation in bracket-

| **CYCLONIC STORMS** | | | |
| --- | --- | --- | --- |
| Agricultural output (cost) | Assets and amenities (cost) | Livestock and Fishery (cost) | Health (cost) |
|  |  |  |  |
|  |  |  |  |
|  |  |  |  |
|  |  |  |  |
|  |  |  |  |

- 1. Compensation Received

4.2.1 Have you received any compensation from the government agencies towards losses incurred due to cyclonic storms? If yes, please provide details.

| Yes/ No |
| --- |
| If Yes, please provide details: |
|  |
|  |
|  |
|  |
|  |

4.2.2 If you have insurance,

| a) Approximate % of the losses from cyclonic storms were covered by your insurance | _________% |
| --- | --- |
| (b)Approximate % of the losses from cyclonic storms were covered by compensations received: | ------------% |
